# Supplementary material for: The causal relationship between the human gut microbiota and pyogenic arthritis: a Mendelian randomization study
Source: Front Cell Infect Microbiol. 2024 Nov 26;14:1452480. doi: 10.3389/fcimb.2024.1452480 (PMC11629706; doi:10.3389/fcimb.2024.1452480)
Supplement: Supplementary file 1 [file DataSheet1.zip › mendelian test/ebi-a-GCST90027521.csv_forest.pdf]

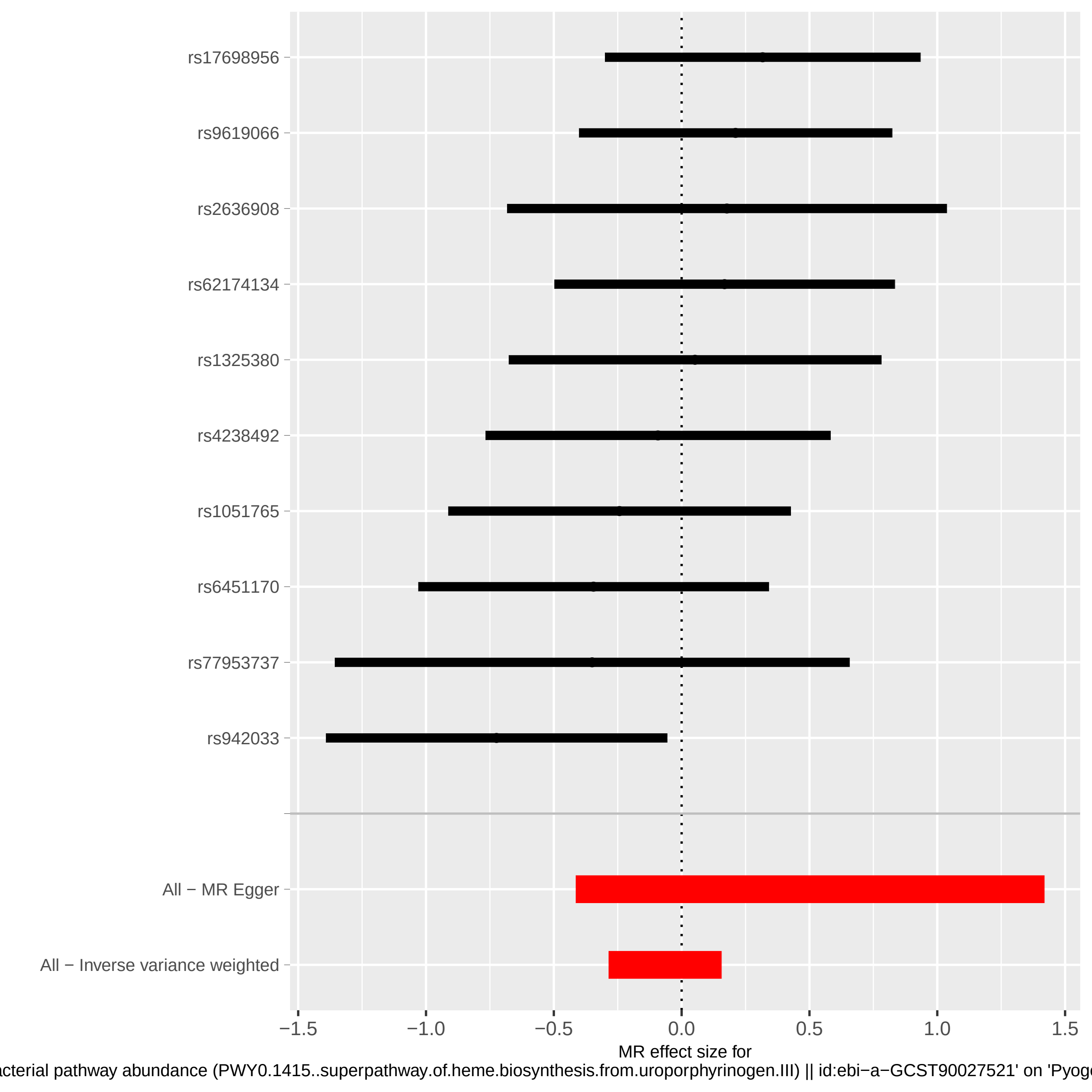
rs17698956
rs9619066
rs2636908
rs62174134
rs1325380
rs4238492
rs1051765
rs6451170
rs77953737
rs942033
All − MR Egger
All − Inverse variance weighted
−1.5
−1.0
−0.5
0.0
0.5
1.0
1.5
MR effect size for
acterial pathway abundance (PWY0.1415..superpathway.of.heme.biosynthesis.from.uroporphyrinogen.III) || id:ebi−a−GCST90027521' on 'Pyog
